# Items

**Published:** 1906-07

**Authors:** 


### ITEMS.

MESSRS. M. J. Breitenbach Company, 53 Warren Street, New York, sometime ago published a bacteriological wall chart suitable for the offices of physicians. It is produced in colors and presents in a most artistic, even classic, manner sixty different forms of pathogenic microorganisms that can be recognized and studied from this chart. It will be sent free to any regular practising physician who, on applying to the publishers, will mention this Journal.

MESSRS. Battle & Company, Saint Louis, announce the issuance of number ten in the series of their twelve illustrated articles on intestinal parasites. These papers will be furnished free to physicians on application, by mentioning this Journal.

